# Effectiveness of preventive interventions on adolescents’ depression and suicidal tendency: a systematic review of randomized controlled trials

**DOI:** 10.3389/fpsyg.2025.1356816

**Published:** 2025-05-05

**Authors:** Lubna Ghazal, Naixue Cui, Cao Fenglin, Erika Sivarajan Froelicher, Mulugeta Shegaze Shimbrer

**Affiliations:** ^1^School of Nursing and Rehabilitation, Cheeloo Medical College, Shandong University, Jinan, China; ^2^Shaukat Khanum Memorial Cancer Hospital and Research Centre, Lahore, Pakistan; ^3^School of Nursing and Rehabilitation, Cheeloo Medical College, Shandong University, Jinan, China; ^4^Research and International Affairs at Cheeloo Medical College, Shandong University, Jinan, China; ^5^School of Medicine, University of California San Francisco, San Francisco, CA, United States; ^6^School of Public Health, Cheeloo College of Medicine, Shandong University, Jinan, China; ^7^School of Public Health, College of Medicine and Health Science, Arba Minch University, Arba Minch, Ethiopia

**Keywords:** adolescents, depression, suicide, children, prevention, promotion

## Abstract

**Introduction:**

This study was conducted to review the effectiveness of preventive mental health interventions for adolescents (aged 11–19 years) in reducing depression and suicidal tendencies.

**Methods:**

A systematic search was conducted to identify randomized controlled trials (RCTs) from the databases *PubMed, CINAHL, EMBASE,* and APA PsycINFO for the period 2011–2024. Studies were included based on strict inclusion/exclusion criteria, selecting those that reported preventive interventions addressing depression and suicidal tendencies among adolescents. The systematic review registration number is CRD42023384321.

**Results:**

Out of 1,210 studies, 13 RCTs were incorporated. The interventions yielded varied outcomes, with approximately half of the RCTs demonstrating reductions in depression and suicidal tendencies, comparable to the control group. Post-intervention, the majority of interventions exhibited mild to moderate effect sizes; however, further research is warranted to assess their long-term efficacy. Cognitive-behavioral therapy (CBT)-based psychoeducation interventions served as the primary approach, often implemented in school settings. However, these interventions were primarily delivered by specialists rather than teachers. Parental involvement in treatment emerged as a potential factor that could enhance the effectiveness of preventive interventions.

**Discussion and conclusion:**

While numerous interventions in this review showed effects comparable to control groups, the diversity in methodology, intervention types, and outcome measures poses challenges in drawing definitive conclusions. Therefore, future research should prioritize addressing these methodological discrepancies within their respective contexts.

## 1 Introduction

Depression and suicidal tendencies are common and significant contributors to adolescent mental health challenges. Globally, one in seven (14%) 10–19-year-olds experiences a mental disorder, accounting for 13% of the global disease burden in this age group (World Health Organization, 2021a). Adolescence is a pivotal phase for sustaining mental well-being, with approximately 50% of adolescents experiencing mental health issues, often emerging around the age of 14 years (NHS, 2022). The prevalence of depression among high schoolers has increased over the past decade. In 2021, over 40% of students reported persistent feelings of sadness or hopelessness, with cumulative data indicating that up to 20–50% of high school adolescents experience at least one clinically depressive episode by the age of 18 years (World Health Organization, 2020). Among adolescents between 15 and 19 years, suicide is the second leading cause of death among girls (after maternal and reproductive issues) and the third leading cause of death among boys (after road traffic accidents and interpersonal violence) (Heidari et al., 2024; World Health Organization, 2021b). The World Health Organization (WHO) report on suicide prevention further highlighted that more than 20% of adolescents have seriously contemplated suicide, while 10% of them have attempted suicide during that period (World Health Organization, 2021b). The alarming prevalence highlights the urgent need for intervention, as one in four adolescents faces mental health challenges requiring immediate attention (Hamdani et al., 2021). Moreover, it is well-documented that many mental health disorders emerge in late childhood and early adolescence, further exacerbating their impact during youth and into adulthood (World Health Organization, 2021a).

Research has established that depression significantly increases the risk of suicide (Hawton and van Heeringen, 2009). Interventions targeting depression, such as cognitive-behavioral therapy (CBT) and resilience-building, inherently address both depressive symptoms and suicide risk. Among adolescents, CBT and antidepressants have been found to be the gold standard treatments for depression and suicidal tendencies. However, the effectiveness of CBT is generally mild to moderate in adolescents. Furthermore, access to CBT can be challenging for adolescents, as it requires qualified health professionals and may not be cost-efficient (Kooistra et al., 2019; Ross et al., 2019). The use of antidepressants remains controversial for adolescents due to side effects, potential dependency, and, most importantly, the overall acceptability among adolescents and their families. Moreover, determining the appropriate dosages and duration of treatment has not consistently led to clinically significant outcomes in many studies (Boaden et al., 2020). These limitations of gold-standard treatments prompt researchers to seek preventive strategies to address depression and suicidal tendencies among adolescents (Ryan and Oquendo, 2020). Preventive interventions appear to be more acceptable and cost-effective options for adolescents and their parents in addressing mental health issues in this population (Singh et al., 2022). Moreover, preventive interventions for adolescents align with the principles of beneficence and non-maleficence, ensuring strategies that promote well-being and minimizing harm.

Effective depression and suicide prevention interventions for adolescents are vital, requiring diverse approaches tailored to their developmental stages. In 2020, Cilar et al. reviewed 57 articles and found that over half of the studies on preventive interventions for adolescents focused on positive psychology and mindfulness, showing positive outcomes (Cilar et al., 2020). However, only four of these studies were considered high quality, indicating a significant gap in robust evidence. This finding highlights the need for high-quality research to validate the effectiveness of these interventions. Key components of successful programs included the settings in which the interventions were implemented. In 2014, Fazal Minai et al. analyzed interventions across various economic settings, examining 42 studies in low-income countries and another 42 in high-income countries (Fazel et al., 2014a; Fazel et al., 2014b). These reviews highlight the crucial role of school-based settings, which are valued for their accessibility and the significant time adolescents spend in them. Similarly, in 2016, Das et al. (2016) conducted a comprehensive umbrella review that included 36 studies, emphasizing the effectiveness of these settings in enhancing mental health and educational outcomes through interventions integrated at both classroom and student levels. Moreover, parental involvement (Flouri and Buchanan, 2002; Williamson et al., 2022) and teacher participation (Franklin et al., 2017; Das et al., 2016) significantly enhance the effectiveness of interventions. However, these approaches also reveal limitations, such as variability in outcomes influenced by socioeconomic contexts and the nature of parental and teacher involvement. Additionally, while teacher involvement is critical for the successful implementation of these programs, a 2019 review by Arora et al., which included 21 studies, emphasized the importance of mental health specialists over school staff in delivering effective interventions (Arora et al., 2019). This distinction often leads to lower effect sizes when non-specialists conduct interventions, highlighting the need for specialized training and consistency in intervention methodology and delivery.

Existing systematic reviews exhibit several limitations, particularly regarding their narrow focus on interventions aimed at reducing symptoms of depression and suicidal tendencies among adolescents aged 13 to 19 years (Singh et al., 2022). Moreover, the evidence frequently lacks specificity regarding participant characteristics, encompassing a broad age range from pre-adolescents (10 years old) to post-adolescents (up to 30 years old). This broad age range can dilute the applicability of findings specifically to adolescents. During this crucial developmental phase, adolescents face complex challenges in fulfilling their biopsychosocial and physical needs without distress. Characterized by significant identity exploration, shifts in social dynamics, and increased academic and societal pressures, these years significantly amplify adolescents’ vulnerability to depression and suicidal tendencies (Skeen et al., 2019). By evaluating the effectiveness of various preventive interventions through randomized controlled trials, this review seeks to determine which interventions are most effective in mitigating these mental health challenges, specifically for the teen population (Richter et al., 2022). The findings of this review will help identify age-specific interventions that can effectively address the unique mental health needs of adolescents.

Second, previous reviews included both clinical and non-clinical populations, resulting in considerable variability in the results (Das et al., 2016; Fazel et al., 2014a; Fazel et al., 2014b; Richter et al., 2022). Thus, it remains unclear how effectively depression and suicidal tendencies among non-clinical adolescents can be prevented or reduced through early interventions. Third, previous research confirms that family members, especially parents, can mitigate adolescents’ depression, feelings of isolation, anxiety, and suicidal inclinations (Kolaski et al., 2024; Van Voorhees et al., 2009). Consequently, the present review aims to investigate the enabling factors for involving parents in interventions. Finally, there exists a gap in the existing literature regarding the effectiveness of intervention delivery by non-specialists compared to specialists. Thus, it is imperative to explore whether these preventive interventions can be effectively and conveniently delivered by non-specialists. Implementing preventive interventions for depression and suicidal tendencies in adolescents could be a challenging task for mental health professionals or specialists due to their limited availability, making access to mental health care difficult due to the scarcity of mental health resources.

The current systematic review aimed to evaluate the effectiveness of preventive interventions reported in the literature, focusing on their impact on reducing depression and suicidal tendencies among adolescents. Additionally, the systematic review analyzed the setting and interventionists (specialist vs. non-specialist) involved in these interventions to identify accessible, cost-effective, and operationally successful interventions for this unique population. Furthermore, the review explored the role of family in mitigating depression and suicidal tendencies among adolescents.

## 2 Method

### 2.1 Protocol and registration

The systematic review protocol was registered and published with the International Prospective Register of Systematic Reviews (PROSPERO) (registration number: CRD42023384321). The review utilized the Preferred Reporting Items for Systematic Reviews and Meta-Analyses (PRISMA) guidelines for reporting the findings (See Supplementary file 1).

### 2.2 Eligibility criteria and exclusion

**Population (P):** Studies involving participants aged 11 to 19 were included, covering a broad adolescent range from pre-adolescents (11–12 years) to adolescents (13–19 years). There were no geographical restrictions, encompassing studies worldwide to ensure a comprehensive understanding of preventive mental health interventions across diverse cultural contexts.

**Interventions (I):** The interventions assessed in the reviewed studies targeted preventive mental health interventions to address depression and suicidal tendencies among adolescents. These interventions encompassed a range of approaches, such as psychotherapy, CBT, mindfulness-based techniques, group therapy, educational programs, and other structured interventions, aimed at enhancing mental well-being and alleviating depressive symptoms and suicidal tendencies among adolescents.

**Comparator (C)**: The review examined randomized controlled trials (RCTs) that compared intervention groups to control groups. Control groups may consist of no treatment, standard care, or an alternative intervention.

**Outcomes (O):** The primary outcomes considered included measures of depression and suicidal tendencies. The secondary outcomes included changes in mental well-being, reductions in depressive symptoms, and the mitigation of suicidal behavior.

**Exclusions:** Publications in languages other than English, pilot studies, study protocols, reviews, commentaries, and letters to the editor. Additionally, studies specifically addressing post-COVID-19 depression, psychotic depression outcomes, and hospital-based treatments were omitted.

The exclusion of non-English publications stemmed from the review team’s linguistic limitations and the costs associated with translation services, leading to a potential language bias. This decision aimed to maintain the review’s feasibility within available resources while acknowledging the bias limitation. To address this limitation, a comprehensive search of English-language databases known for their extensive international research coverage was conducted.

Although valuable in the early intervention stages, pilot and feasibility RCTs were excluded because they focus more on procedural feasibility than on efficacy outcomes and often lack the statistical power needed for reliable efficacy data. This review focused on studies that were sufficiently robust to provide conclusive data on the efficacy of preventive mental health interventions for adolescents.

Studies on post-COVID-19 and psychotic depression were excluded due to their distinct etiological mechanisms and specialized treatment requirements. Including them could compromise the generalizability of our findings, as these conditions demand tailored interventions rather than the broad preventive strategies analyzed for general depressive populations. The focus was on non-hospital settings to emphasize interventions accessible in community or school environments, aligning with the review’s aim to explore preventive strategies rather than treatments for severe conditions necessitating acute care (Supplementary file 2).

### 2.3 Literature search strategy

**Figure 1:**
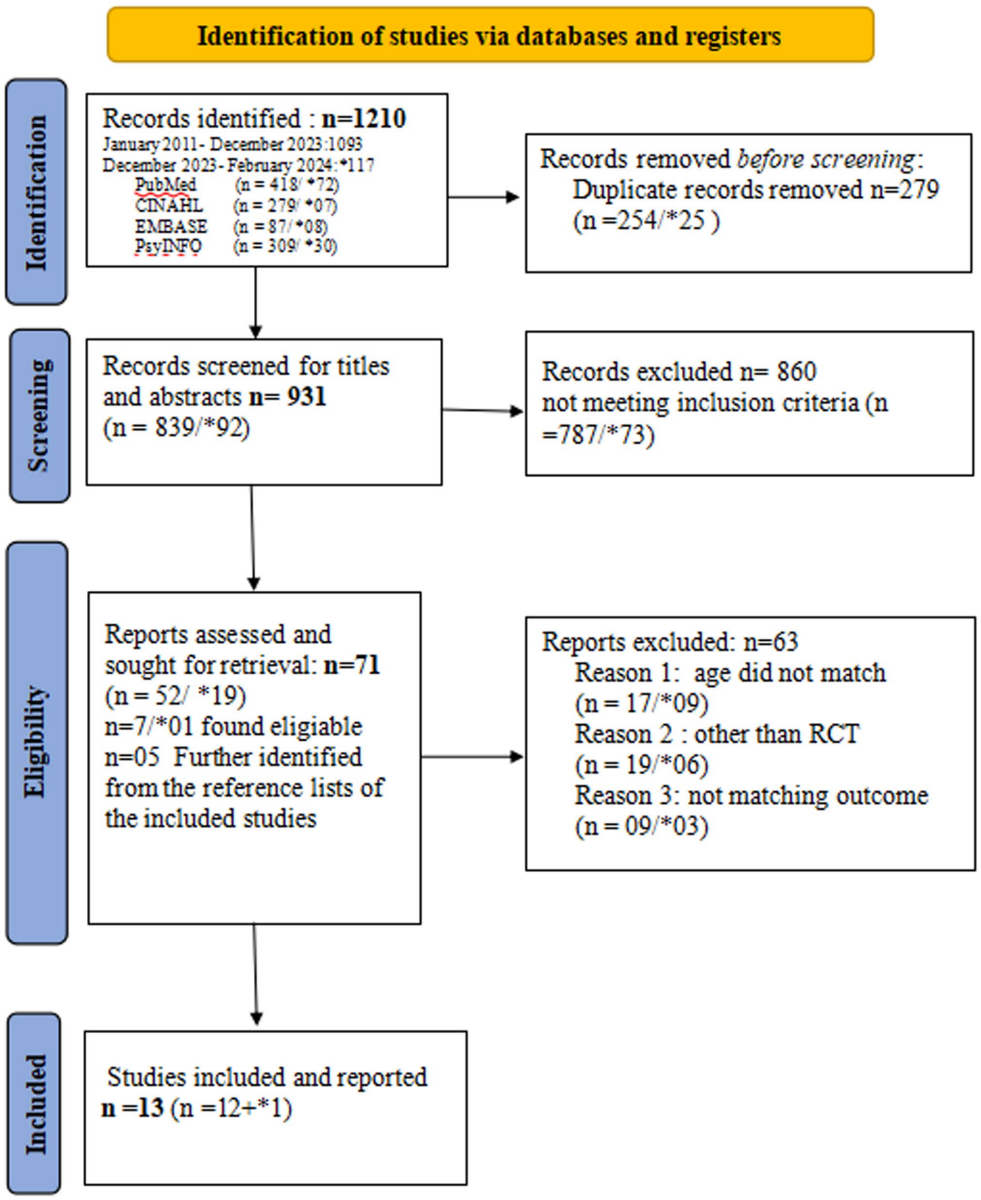
The preferred reporting items for systematic reviews and meta-analyses (PRISMA) 2020. The asteric (*) in the figure shows data related to revised search upto February 2024.

The review employed a systematic literature search strategy to identify relevant studies on mental health interventions aimed at reducing depression and suicidal tendencies in adolescents. The search encompassed databases including PubMed, CINAHL, Embase, and APA PsycINFO from 1 January 2011 to 15 December 2023 (*n* = 1,093). To update the review with recent literature, a revised search was conducted from 16 December 2023 to 23 February 2024, yielding 117 citations (Supplementary file 3). We selected 1 January 2011 as the starting point for our literature search to focus on the most recent evidence reflecting advancements. This date corresponds with a notable shift in the availability of high-quality RCTs identified during a preliminary review. Spanning a decade, this timeline facilitates a comprehensive analysis of contemporary evidence. Previous seminal reviews (Das et al., 2016; Fazel et al., 2014a; Fazel et al., 2014b) have addressed research prior to 2011. To prevent overlap and build on this existing knowledge base, we focused on newer studies while also reviewing the references of earlier works to include any crucial prior research. Figure 1 illustrates the updated search process, from database querying to the final inclusion of citations, as indicated by an asterisk in the PRISMA Flow diagram.

### 2.4 Studies selection

By utilizing Colandr, a web-based open-access reference manager, we systematically stored, screened, and shortlisted studies based on predefined inclusion and exclusion criteria. From the identified 1,210 records, we removed 279 duplicates, leading to the review of 931 citations. During the title and abstract screening, we excluded 860 studies that did not meet the preset criteria. Two independent reviewers assessed the titles and abstracts, resulting in the shortlisting of 71 articles for full-text screening. Following this comprehensive process, 13 studies were ultimately included in the review. The PRISMA flow diagram (See Figure 1) details the process through which the studies were identified, screened, and included in this review. The full texts of the included citations were retrieved and saved in Colandr for further data extraction, while the excluded studies were coded to indicate the reason for their exclusion. Any disagreements regarding the selection of citations between the two primary reviewers were resolved by a third reviewer and research advisors.

### 2.5 Data extraction

Upon the identification of eligible studies, the first and second authors collaborated to prepare a literature summary table that facilitated the systematic data extraction for review. Two reviewers then independently utilized this summary table to meticulously extract relevant information from each study, ensuring a solid foundation for our analysis. This comprehensive data extraction process included authors, country of origin, year of publication, quality appraisal score, sample size, gender, age, setting, specifics of the intervention, follow-up intervals, employed tools/instruments/measures, primary outcomes, and data regarding effect estimates and the intervention’s effectiveness compared to a control group, as well as statistical significance.

In addition to these critical elements, special attention was given to the extraction of effect size data, including reported effect sizes (e.g., Cohen’s *d*, correlation coefficients), means, standard deviations, and confidence intervals. This approach allowed for a uniform assessment of the interventions’ effectiveness across studies, accommodating the diverse reporting formats found in the literature. In cases where effect sizes were not explicitly reported, such as in studies providing only means and standard deviations, Cohen’s *d* was calculated to standardize the effect size measurement across studies. This approach was exemplified by our treatment of specific studies such as Bernal et al. (2019), which reported results using correlation coefficients, thereby facilitating effect size calculation. This detailed and methodical extraction process fostered a nuanced understanding of intervention effectiveness, ensuring a comprehensive and insightful analysis (Bernal et al., 2019).

### 2.6 Critical appraisal: JBI

For this systematic review, the assessment of RCTs was rigorously conducted using the Joanna Briggs Institute (JBI) tools, which are widely recognized in academic circles for their robustness and validity in evaluating research quality (Ma et al., 2020). Two independent reviewers meticulously carried out the evaluation process, analyzing each study based on its objectives, sampling strategy, measurement accuracy, and analytical robustness.

The assessment framework included a comprehensive risk of bias analysis, detailed across 13 specific criteria (Supplementary file 4). These criteria covered five critical domains of RCT methodology: the randomization process, adherence to intended interventions, management of missing outcome data, accuracy of outcome measurement, and selection of reported results. Each criterion was assigned a point, with the total score providing a quantifiable measure of the study’s methodological soundness. A higher score indicated superior research quality (Porritt et al., 2014; Ma et al., 2020). In instances of disagreement, reviewers reached consensus through thorough discussion, ensuring a balanced and objective quality appraisal.

### 2.7 Data synthesis and analysis

In this systematic review, the primary goal was to synthesize evidence regarding the effectiveness of preventive interventions, as reported in the studies. Among the 13 studies examined, only three directly reported the effect size of their intervention. Of the remaining studies, five reported the significance of results using means, standard deviations, and confidence intervals, necessitating the computation of Cohen’s *d* to standardize the measure of intervention effectiveness. Specifically, Bernal et al. (2019) utilized a correlation coefficient for effect size calculation, highlighting the varied methodologies employed across the research landscape. Given the significant variation in follow-up time points for outcome measurement across studies and the prevalence of selective data reporting, a strategic decision was made to compute effect sizes at four critical time points: baseline, post-intervention, mid-point, and end-point. This approach facilitated a more granular analysis of intervention effectiveness over time. For instance, Bernal et al. (2019) conducted a 12-week intervention with six follow-up time points at 3, 6, 9, 12, and 15 months, enabling the computation of effect size at 3 months (post-intervention), 9 months (mid-point), and 15 months (end-point) (Bernal et al., 2019), as detailed in Supplementary file 5.

The significant heterogeneity among the studies—regarding outcome measures, intervention types, and follow-up periods—prevented the feasibility of conducting a meta-analysis. Instead, a narrative synthesis approach in systematic reviews, was employed. This methodology allowed for the systematic evaluation and integration of findings from the diverse range of studies included in the review. In doing so, it provided substantive insights into the effectiveness of various preventive interventions. The narrative synthesis approach proved particularly advantageous given the complexity of the review, allowing for a thorough exploration of the data while carefully navigating the inherent heterogeneity in study designs, active controls, outcome measures, and reporting practices encountered.

Thus, the strategic computation of Cohen’s d and, in cases such as that of Bernal et al. (2019), the use of correlation coefficients for effect size calculation were integral to the analytical strategy. These calculations enriched the synthesis by providing a quantifiable measure of intervention effectiveness where possible, demonstrating a commitment to a rigorous, yet flexible, examination of the evidence. This nuanced approach, aimed at identifying effective strategies for reducing depression and suicidal tendencies among adolescents, elevates the review beyond mere descriptive summarization, offering a comprehensive and insightful analysis of preventive mental health interventions.

## 3 Results

### 3.1 Study selection

The PRISMA flow diagram (Refer to Figure 1) illustrates the study selection process. All selected studies were RCTs. These studies examined the effectiveness of at least one preventive intervention compared to a control group, with a focus on reducing depression and suicidal tendencies among adolescents as outcome measures.

### 3.2 Assessment for risk of biases

The JBI tool for RCTs, as outlined in the Supplementary material, was utilized to assess the quality of each study included in this review. The majority of these studies achieved a score of 85% (10/13). Notably, 10 of the 13 studies encountered challenges related to the blinding of participants and treatment providers, which resulted in lower scores. This is a frequent issue in studies involving psycho-educational interventions, highlighting the inherent difficulty of achieving blinding in behavioral or psycho-educational research (See Supplementary file 4).

### 3.3 Study characteristics

This systematic review included 13 RCTs targeting depression and suicidal tendencies among adolescents (Refer to Supplementary Table 1). These studies were conducted across various developed countries, with four studies conducted in the USA (Bernal et al., 2019; Diamond et al., 2019; Saulsberry et al., 2013; Schwartz et al., 2023), two in New Zealand (Merry et al., 2012; Whittaker et al., 2017), two in Norway (Waraan et al., 2021a; Waraan et al., 2021b), and one each in Australia (Hetrick et al., 2017), Canada (Silverstone et al., 2017), the UK (Stallard et al., 2013), the Netherlands (de Jonge-Heesen et al., 2020), and Japan (Nagamitsu et al., 2022). The total sample comprised 10,707 adolescents aged 11 to 19 years, with a significant proportion of women (40%). The studies vary widely in sample size, ranging from 50 (Hetrick et al., 2017) to 5,030 (Stallard et al., 2013), indicating diverse research scopes and settings (Refer to Table 1 for key findings).

**Table 1:** Key findings.

Theme	Key findings	Number of studies reporting this finding
Intervention effectiveness	More than half found statistically significant reduction in depression or suicidal tendencies on three months follow-up	7/13
Population characteristics	Studies included adolescents aged 11-19, mostly 13-19	6/13
Study designs	All studies	13/13
Common measures used	Measurement tools varied widely	–
Studies’ origin	Studies originated from high-income countries	13/13

### 3.4 Settings, intervention modalities, and format

Interventions were predominantly conducted in school settings (de Jonge-Heesen et al., 2020; Hetrick et al., 2017; Nagamitsu et al., 2022; Silverstone et al., 2017; Stallard et al., 2013; Whittaker et al., 2017), underscoring their accessibility and the potential for integrated mental health support in educational environments. Community clinics were used in five studies (Bernal et al., 2019; Waraan et al., 2021a; Waraan et al., 2021b; Saulsberry et al., 2013; Schwartz et al., 2023), highlighting their role in targeted intervention delivery. Additionally, two studies utilized multiple settings, including schools, community clinics, and emergency rooms, to broaden demographic reach (Diamond et al., 2019; Merry et al., 2012).

Each study employed different intervention strategies, with a common focus on structured, manualized approaches. Predominantly, face-to-face delivery was employed in six studies (Bernal et al., 2019; de Jonge-Heesen et al., 2020; Diamond et al., 2019; Schwartz et al., 2023; Stallard et al., 2013; Waraan et al., 2021a; Waraan et al., 2021b), allowing therapists and participants to engage directly, which is crucial for effective mental health interventions. Additionally, four studies integrated digital platforms with traditional face-to-face methods (Hetrick et al., 2017; Nagamitsu et al., 2022; Saulsberry et al., 2013; Silverstone et al., 2017), enhancing accessibility and engagement through modern technology. Three studies adopted a fully digital approach, utilizing web-based platforms, mobile apps, and gaming to deliver interventions (Merry et al., 2012; Whittaker et al., 2017), demonstrating innovative strategies to address teen mental health in the digital era. These diverse methods reflect the evolving landscape of mental health interventions, adapting to both traditional and contemporary modalities to better meet the needs of adolescents. Intervention lengths ranged from 2 weeks (Nagamitsu et al., 2022) to 16 weeks (Diamond et al., 2019), with session durations adjusted for target populations, varying between 40 and 120 min. Specialists, including psychologists, physicians, and social workers, primarily implemented these interventions to ensure professional oversight and consistency.

### 3.5 Comparison

Nine studies utilized active controls to benchmark the interventions (Bernal et al., 2019; de Jonge-Heesen et al., 2020; Diamond et al., 2019; Saulsberry et al., 2013; Schwartz et al., 2023; Stallard et al., 2013; Waraan et al., 2021a; Waraan et al., 2021b; Whittaker et al., 2017), with comparisons ranging from standard care practices to more structured psychotherapeutic approaches such as CBT. Two studies used treatment-as-usual as a control (Hetrick et al., 2017; Merry et al., 2012), providing a baseline for evaluating the added value of the interventions. This diverse array of control conditions facilitates a robust assessment of intervention effectiveness across different therapeutic environments and methodologies.

### 3.6 Outcome and follow-up time points

The primary outcomes targeted by the interventions varied across the 13 studies, focusing on the effectiveness of preventive interventions against depression and suicidal tendencies among adolescents. Seven studies specifically reported depression as their primary outcome (de Jonge-Heesen et al., 2020; Merry et al., 2012; Nagamitsu et al., 2022; Saulsberry et al., 2013; Stallard et al., 2013; Waraan et al., 2021a; Waraan et al., 2021b; Whittaker et al., 2017), three studies examined the impact on suicidal tendencies (Hetrick et al., 2017; Silverstone et al., 2017; Waraan et al., 2021a; Waraan et al., 2021b), and three others addressed both depression and suicidal tendencies within their analyses (Bernal et al., 2019; Diamond et al., 2019; Schwartz et al., 2023).

There was also significant variability in the frequency and duration of outcome assessments across these studies, reflecting the complexity of measuring the long-term effects of mental health interventions. The follow-up periods ranged from two to five time points (up to 15 months), with some studies, such as those by Bernal et al. (2019) and Silverstone et al. (2017), providing more extensive longitudinal data to better understand the sustainability of intervention effects. Notably, Diamond et al. (2019) focused exclusively on post-intervention outcomes, underscoring the immediate impact of their intervention. The variations in follow-up assessments across these studies highlight the challenges in synthesizing the effectiveness of interventions and demonstrate the need for consistent measurement approaches to reliably gauge their long-term benefits.

### 3.7 Effectiveness of the interventions

In evaluating the outcomes of 13 selected studies, it was revealed that seven reported reductions in depression and suicidal ideation among adolescents following the interventions, lasting up to 3 months. Despite these positive findings, a direct comparison did not consistently demonstrate that these interventions outperformed usual care or control groups. Meanwhile, two studies, Nagamitsu et al. (2022) and Silverstone et al. (2017), highlighted the effectiveness of their interventions compared to control conditions, while Merry et al. (2012) reported outcomes similar to those of usual care.

Only three of the 13 studies provided effect size data (de Jonge-Heesen et al., 2020; Diamond et al., 2019; Schwartz et al., 2023). For the remaining 10 studies, effect size was computed (Please refer to the “Methods” section and Supplementary file 5). The analysis revealed varied outcomes: While the majority of interventions did not show a significant advantage over control or treatment-as-usual (TAU) groups, exceptions were noted. Specifically, three studies (Nagamitsu et al., 2022; Schwartz et al., 2023; Silverstone et al., 2017) highlighted the efficacy of their interventions beyond that of their respective controls. In contrast, one study (Merry et al., 2012) reported that its intervention achieved results comparable to TAU, underscoring the potential for certain approaches to match the effectiveness of existing care protocols. In addition, the majority of evaluated interventions yielded minimal to small effect sizes, indicating a modest immediate impact on depression and suicidal ideation.

On the other hand, the impact of interventions on suicidal ideation and tendencies presents a complex picture, with studies demonstrating a range of outcomes. For example, Silverstone et al. (2017) and Diamond et al. (2019) explored interventions that included significant parental involvement and reported a notable reduction in suicidal tendencies among participants. Silverstone et al. (2017) implemented the EMPATHY program, which combined face-to-face and online CBT elements, showing a moderate effect on reducing suicidal ideation. Diamond et al. (2019)‘s approach, integrating Attachment-Based Family Therapy (ABFT) with parental coaching, yielded one of the highest effect sizes for reducing both depression and suicidal tendencies, highlighting the added value of family involvement in treatment.

### 3.8 Potential factors of effectiveness

#### 3.8.1 Settings

The effectiveness of interventions varied significantly across different settings. Eight studies focused on school settings (Diamond et al., 2019; Nagamitsu et al., 2022; de Jonge-Heesen et al., 2020; Hetrick et al., 2017; Merry et al., 2012; Silverstone et al., 2017), demonstrating pronounced efficacy, with six reporting statistically significant improvements in teen mental health (See Supplementary Table 1). Conversely, five studies conducted in community clinics showed mixed results, with Saulsberry et al. (2013) being the only study to report significant outcomes, highlighting the potential influence of the intervention setting on success rates.

#### 3.8.2 Delivery by specialists versus non-specialists

Specialists, including psychologists and research investigators, conducted the interventions in 11 of the 13 studies, demonstrating a high level of expertise in addressing sensitive mental health issues (Bernal et al., 2019; Diamond et al., 2019; Hetrick et al., 2017; Merry et al., 2012; Nagamitsu et al., 2022; Waraan et al., 2021a; Whittaker et al., 2017). Only three school-based studies involved school personnel for control interventions, highlighting the importance of specialist involvement in achieving impactful results (de Jonge-Heesen et al., 2020; Stallard et al., 2013). Notably, Silverstone et al. (2017) utilized resiliency coaches, showing that targeted expertise in mental health and CBT can be crucial even outside conventional clinical settings.

#### 3.8.3 Parental involvement in interventions

Parental involvement varied across studies. Four studies explicitly involved parents in the interventions, aiming to leverage family dynamics to improve outcomes for adolescents, especially in reducing suicidal tendencies and depression (Bernal et al., 2019; Diamond et al., 2019; Waraan et al., 2021a; Waraan et al., 2021b). Diamond et al. (2019) showed strong efficacy with parental involvement, indicating the potential benefits of a family-centered approach. However, studies with minimal parental involvement or only parental monitoring also reported favorable effects, suggesting that multiple approaches can be effective depending on the intervention’s focus and setting (de Jonge-Heesen et al., 2020; Schwartz et al., 2023; Silverstone et al., 2017).

#### 3.8.4 Cost-effectiveness of the intervention

Among the 13 studies, only Stallard et al. (2013) conducted a cost analysis, concluding that interventions were more expensive than controls (intervention costs per child: classroom-based CBT £41.96 and attention control PSHE £34.45). Their study’s intervention costs included facilitators’ fees for delivering the intervention, training expenses, travel expenses, printing of training manuals, and participant referral and treatment expenses (Stallard et al., 2013). The remaining studies 12 studies did not perform a comprehensive cost analysis. However, all studies acknowledged receiving funding to conduct the trial, indicating that mental health preventive interventions require a financial investment for successful implementation.

## 4 Discussion

### 4.1 Effectiveness of the preventive interventions

Many of the examined interventions were found to have effects equal to or less than those of their control counterparts in reducing depression and suicidal tendencies among adolescents. This variability in outcomes can be attributed to several factors: small sample sizes with attrition rates exceeding 20% and significant missing data (Asarnow and Miranda, 2014), interventions not being sufficiently tailored to meet the unique developmental and emotional needs of adolescents (Ragelienė, 2016; Weersing and Brent, 2006), and the influence of the provider’s competence, available resources, and the context of delivery (Cuijpers et al., 2016; Purgato et al., 2020). While many interventions target both depression and suicidal tendencies, it is important to recognize that these two conditions may require different intervention approaches. Depression-focused interventions often prioritize mood regulation, cognitive restructuring, and coping strategies (Cuijpers et al., 2014), whereas interventions targeting suicidal tendencies may emphasize crisis management, safety planning, and immediate risk reduction (Zalsman et al., 2016). Although depression is a significant risk factor for suicidal behavior, the pathways leading to suicidal actions can be distinct, necessitating more specialized intervention strategies. Future research should explore these differences in greater detail to better tailor preventive interventions to each condition and improve their effectiveness.

Moreover, using active controls in studies may further obscure the true efficacy of interventions. According to equipoise theory, ethical trial design requires that treatments provided to control groups be as effective as possible, potentially reducing observed differences (Freedman, 1987). Additionally, response expectancy theory suggests that participants’ expectations of receiving beneficial treatment, regardless of group assignment, can enhance outcomes in both the intervention and control groups (Kirsch, 1985). This psychological effect, along with the ethical obligation to provide effective treatment to control groups, may lead to smaller observed differences between intervention outcomes and those of active controls, complicating the assessment of intervention superiority.

Effective intervention requires meticulous implementation and evaluation, tailored to the specific needs of adolescents (Fazel et al., 2014a; Fazel et al., 2014b; Weersing and Brent, 2006). Future research should focus on enhancing adherence strategies and integrating qualitative components to understand the motivations behind participant non-adherence. Additionally, adopting hybrid designs could provide insights into how different implementation strategies affect the overall effectiveness of interventions (Trutschel et al., 2023; Ullman et al., 2022).

### 4.2 Key factors to effectiveness

#### 4.2.1 Study setting

The current review finds that school-based interventions are notably more effective than those delivered in community clinics. This observation is supported by Social Ecological Theory, which emphasizes the central role of schools in adolescents’ lives and their multilevel influence on behavior (Hardcastle et al., 1981). Schools provide structured, consistent settings that enhance adherence to interventions and their overall effectiveness, distinguishing them from the less effective settings in community clinics. Moreover, logistical challenges often hinder adolescents’ participation in community clinic treatments due to the requirement for adult accompaniment, which aligns with the Theory of Planned Behavior that suggests perceived control significantly influences behavioral intentions (Ajzen, 1991). Additionally, Disruption Theory posits that the lengthy duration of these programs may disrupt adolescents’ routines, further reducing engagement (Christensen, 2013) in community clinics. Furthermore, Stigma Theory explains that the stigma surrounding mental health can deter families from seeking community services, whereas schools provide a stigma-free environment that promotes regular participation (Goffman, 1964). Given these insights, schools are increasingly recognized as optimal venues for mental health interventions, suggesting that future research should prioritize these settings, particularly in low- and middle-income countries (Magis-Weinberg et al., 2021; Nagata et al., 2018; Van Voorhees et al., 2009).

#### 4.2.2 Delivery of intervention specialist versus non-specialist

This systematic review reveals that, while specialist-delivered interventions are effective, they may not be sustainable due to high costs. Task shifting to non-specialists, such as teachers in schools, shows promise for improving mental health affordably, particularly in low-resource settings (Coates and Foureur, 2019; Evans et al., 2020; Imran et al., 2022). Supported by the Diffusion of Innovations Theory (Rogers, 2003) and Social Learning Theory (Bandura, 1977), this approach leverages the integral role of schools and the influence of teachers to enhance sustainability and effectiveness. The Community-Based Participatory Research framework further justifies the involvement of teachers to increase the relevance and impact of interventions. Despite promising preliminary results, the lack of RCTs utilizing teachers highlights a critical area for future research to explore and validate the effectiveness of such interventions.

#### 4.2.3 Parental involvement

Parental involvement in interventions is crucial during the sensitive developmental stage of adolescence, as up to 50% of adolescents may develop mental disorders by the age of 14 years (Fusar-Poli et al., 2019). Although existing literature highlights the benefits of family engagement in reducing depression, isolation, and suicidal tendencies among adolescents (Brady et al., 2016), the studies reviewed showed that including parents did not significantly outperform control groups or standard treatments. This lack of enhanced effectiveness could be attributed to high parental attrition rates, impacted by busy schedules, cultural or language barriers, and socioeconomic constraints (Martins et al., 2020). Future research should focus on improving parental enrollment and adherence strategies while providing detailed participation metrics to better understand and enhance the efficacy of family-involved interventions.

#### 4.2.4 Cost-effectiveness of the intervention

Only one study in this review evaluated cost-effectiveness, highlighting the need for economical interventions, especially in low- and middle-income countries (LMICs), where resources are limited (Fazel et al., 2014a; Fazel et al., 2014b). UNICEF (2022) advocates for investment in school-based psychosocial programs that offer a favorable benefit-to-cost ratio (UNICEF, 2022). Cost reductions and enhanced effectiveness can be achieved by leveraging existing school resources, such as teachers and counselors, and integrating community-based mental health services, which are less costly than new in-school services. Furthermore, a tiered support system ensures that all students receive some level of support, with more intensive care provided to those with greater needs, optimizing resource use and intervention impact. Future research must prioritize cost-effectiveness to maximize the reach and sustainability of mental health interventions.

## 5 Strengths and limitations

This review was conducted in an objective and well-structured manner, adhering rigorously to predefined criteria with prospective registration on PROSPERO. Nonetheless, it has limitations. It relied exclusively on English-language literature from electronic databases, potentially overlooking relevant studies published in other languages. The strict age range of 13 to 19 years limited the number of studies included, possibly affecting the perceived comprehensiveness of the review. This limitation suggests a gap in interventions tailored specifically for this age group, highlighting the need for further development in this area. Additionally, the majority of the selected studies originated from the developed world, indicating a lack of diverse evidence from LMICs, which may limit the generalizability of the findings.

## 6 Conclusion

This review sought to identify effective preventive mental health interventions aimed at reducing depression and suicidal tendencies among adolescents. While all included interventions demonstrated some ability to alleviate these issues, they were not found to be superior to controls or standard treatment. The findings emphasize the importance of carefully selecting both the setting and facilitator of the intervention to optimize outcomes. Moreover, the review highlighted the critical role of parental involvement, suggesting that effective methods are needed to enhance parents’ interest and adherence to interventions. There is a pressing need for high-quality, cost-effective research, particularly from resource-limited regions, to better prevent and manage depression and suicidal thoughts in adolescents. Future research should prioritize and refine mental health preventive interventions and outcomes to expand the current knowledge base and improve intervention strategies.

## Data Availability

The datasets presented in this study can be found in online repositories. The names of the repository/repositories and accession number (s) can be found in the article/Supplementary material.
