# Supplementary material for: Effectiveness of preventive interventions on adolescents’ depression and suicidal tendency: a systematic review of randomized controlled trials
Source: Front Psychol. 2025 May 5;16:1356816. doi: 10.3389/fpsyg.2025.1356816 (PMC12087179; doi:10.3389/fpsyg.2025.1356816)
Supplement: Supplementary file 1 [file Table_1.docx]

| **Table 1: Studies’ Characteristics** | | | | | | | | | | | | | | | | |
| --- | --- | --- | --- | --- | --- | --- | --- | --- | --- | --- | --- | --- | --- | --- | --- | --- |
| **Sr #** | **Author &Country** | **JBI** | **Sample size** | **Gender**  **F%** | **Age** | **Setting** | **Intervention &Controls with Sample size** | **Format** | **Delivered by** | **Training** | **Length** | **Follow-up** | **Primary outcome** | **Secondary Outcome** | **Intervention Effectiveness Between Groups (Int vs Cont)** | **Overall Intervention Effectiveness Summary, Between Groups, Across Various Time Points,** |
| **1** | Schwartz  et al., 2023  USA | 11 | 185 | 58 | 11.3 | Clinics | BBT (n=95) | Face to Face | Therapists | Therapist were trained for intervention | 8-12 weekly 45-minute sessions over a 16-week period | 4 & 8 Months | Absence of depression (MFQ) | SDQ-P: | **NSS** | BBT continued to be effective, especially in managing anxiety, yet the degree of its impact on depression and suicidality lessened somewhat as the time extended from 4 to 8 months post-intervention, indicating a diminishing effect for these particular measures. |
|  |  |  |  |  |  |  | ARC Control (n=90) |  |  |  |  |  | Anxiety  (SCARED) |  | **SS** |  |
|  |  |  |  |  |  |  |  |  |  |  |  |  | Suicidality Index (SI) |  | **NSS** |  |
| 2 | Nagamitsu et al., 2022 Japan | 11 | 209 | 62 | 13-18 | Schools - | WCV: well-care visit (n1=67) | Face to Face | Investigators | A training video was used to demonstrate the technique | 2 weeks face-to-face counselling- 40 minutes session | 1, 2 & 4Months | Reduction in depression (DSRS-C) | Suicidal ideation PHQ-9 ; AHP-SF; RSES; TEIQue-ASF | SS for WCV | WCV sustained a moderate effect on depression in teens, while adding a CBT app showed early promise but didn't maintain significant benefits over time. |
|  |  |  |  |  |  |  | WCV: well-care visit & CBT: app. (n2=70) | Combination of well-care clinic visits & use of CBT through Phone app | Smartphone app | Not mentioned | 2 weeks of psychoeducation (week 1) and a self-monitoring session (week 2). |  |  |  | NS for WCV+ Phone App CBT |  |
|  |  |  |  |  |  |  | Wait list Control (n3=72) | face-to-face or Smartphone app | Investigators/Smartphone app | Not mentioned | Same as above |  |  |  |  |  |
| 3 | (A) Waraan et al, 2021, Norway | 10 | 60 | 87 | 13 -18 | Clinics | ABFT:30 | face to face | clinical psychologists trained for the therapy (n-8) | Psychologists trained in ABFT for the purpose of the trial | 16 Weeks  Session duration not mentioned | 4, 6, 12 Months | Clinician-rated GRID HAMD = Hamilton Depression Rating Scale | none | *NSS* | The intervention showed a small to medium effect size (Cohen's d = 0.322), but this did not translate into statistical significance, with a high P-value suggesting no reliable difference from the control. Moreover, the positive direction of the effect size raises questions, as a reduction in depressive symptoms would be indicated by a negative value. This discrepancy suggests the intervention may not have functioned as anticipated |
|  |  |  |  |  |  |  | Active Control: 30 |  |  | Trained psychologist |  |  |  |  |  |  |
| 4 | (B)Waraan et al, 2021, Norway | 10 | 60 | 87 | 13 -18 | Clinics | ABFT:30 | face to face | clinical psychologists trained for the therapy (n-8) | Psychologists rained in ABFT for the purpose of the trial | 16 Weeks  Each Session duration | every  other week from week 4 to the end of treatment at week 16 | SIQ-J | none | *NSS* | The intervention's impact was insignificant, failing to surpass the control group in mitigating suicidal tendencies among teenagers. |
|  |  |  |  |  |  |  | Active Control: 30 |  |  | Trained psychologist | Not mentioned |  |  |  |  |  |
| 5 | de Jonge-Heesen et al., 2020  Netherlands | 10 | 130 | 64 | 12-16 | Schools | OVK 2.0 :( n=66) | face to face | School Psychologists & research team members | A 3 day training programme | 8 weeks, one session / 1 hour | 3, 6 & 12 Months | Teen’ CDI-2 | ADIS-C  CDI-2 ( 8 items) for suicidal tendency | NSS | The intervention (OVK) does not appear to be effective in reducing depressive symptoms among teens according to both their own self-reports and the reports of their parents. From the teen's perspective, the change was not statistically significant, indicating no effective reduction in symptoms. From the parents' perspective, the intervention appears to have led to an increase in observed symptoms. This discrepancy suggests that the intervention's effects might vary depending on the perspective of the reporter, highlighting the importance of considering multiple viewpoints when evaluating the effectiveness of mental health interventions. |
|  |  |  |  |  |  |  | Active Control:  Psycho-education (n=64) |  |  |  | Broucher and emails) |  | Parents ’ CDI-2 |  | NSS |  |
| 6 | Diamond et al, 2019 USA | 12 | 129 | 82 | 12 to 18 | Multi-gated | AFBT (n=66) | Face to Face | 14 trained therapists (MS and PhD level) | Yes | 16 week each Session duration not mentioned | 1, 2, 3 and 4 Months | SIQ-JR, | none | SS | Both ABFT and FE-NST ( active control) produced substantial reductions in suicidal ideation and depressive symptoms. The effect sizes for both treatments were large and clinically significant, indicating that both interventions and active control were highly effective. |
|  |  |  |  |  |  |  | Active Control: FE-NST (n=63) |  |  |  |  |  | BDI |  | SS |  |
| 7 | Bernal 2019, USA | 11 | 121 | 67 | 11-17.5 | Community Clinic | TESPI+CBT: 60 | Face to Face | Psychology PhD students with 3 years of clinical experience trained external instructors according to a manualized curriculum | Yes | 12 weeks/ 12 sessions (individual CBT) TEPSI 8 group sessions each 2 hours | 3,6,9, 12, 15 Months | CDI | BDI-R  FEICS  B-FAM  FS | NSS | TEPSI+CBT intervention does not demonstrate a statistically significant advantage over CBT alone at reducing depressive symptoms across the measured intervals. |
|  |  |  |  |  |  |  |  |  |  |  |  |  | CDRS-R |  | NSS |  |
|  |  |  |  |  |  |  | Active Control-CBT:61 |  |  | _ |  |  | SIQ-Jr |  | NSS |  |
| 8 | Silverstone et al 2017,  Canada | 9 | 1884 | 34 | 11 to 18 | School | EMPATHY: 1884 | F2F and online guided CBT for high-risk group | Resiliency Coaches (specially hired for the program, non-specialist but having experience working with adolescents, | Not clearly mentioned | not mentioned - retrieved from earlier publication -8 weeks | 3,7 and 15-Month | PHQ-9 | None | *SS* | Throughout the 3, 7, and 15-month follow-ups, the EMPATHY program consistently demonstrated effectiveness in reducing symptoms of depression and anxiety, as evidenced by statistically significant small effect sizes. Additionally, the program had a moderate and significant impact on reducing suicidal risk among students, indicating its sustained effectiveness in improving critical aspects of youth mental health over time. |
|  |  |  |  |  |  |  |  |  |  |  |  |  | HAD |  | *SS* |  |
|  |  |  |  |  |  |  | Own Control |  |  |  |  |  | Suicidality (HAD 3 questions) |  | *SS* |  |
| 9 | Hetrick et al, 2017, Australia | 11 | 50 | 82 | 13 to 19 | School | The Reframe-IT intervention Web-based CBT: 26 | F2F +Web based | Registered clinical psychologist | Yes | 10 Weeks | 2.5 and 5.5 months | SIQ | BHS,  MASC,  NPOQ, CBTSQ, CBTSQ-BA, CBTSQ-CR | NSS | The Reframe-IT intervention suggested potential reductions in suicidal ideation and depression at 2.5 and 5.5 months, with small effect sizes. However, the evidence is inconclusive due to wide confidence intervals, non-significant p-values. considering small sample sizes and high dropout rates in this study may compromise the robustness of study findings, potentially reducing statistical power and introducing bias. These issues limit the generalizability of results, suggesting the need for further research with larger, more stable samples to determine the intervention’s effectiveness. |
|  |  |  |  |  |  |  | Control (TAU): 24 | _ | The student  well-being staff member administered the program at school. | _ |  |  | RADS, |  | NSS |  |
|  |  |  |  |  |  |  |  |  |  |  |  |  | CDRS-R |  | NSS |  |
| 10 | Whittaker et al 2017,  New Zealand | 13 | 855 | 68 | 13 to 17 | School | MEMO-CBT: 426 | Mobile phone-based | A trained research assistant | Yes | 9 Weeks | 2 Month 12 Months | Depressive symptoms  Clinician rated (CDRS-R) | Quality of life (PQ-LES-Q) Mood and feeling (MFQ) Suicidal ideation and plan (YRBS) Affective disorder (K-SADS) | NSS | Considering the post intervention 2 and 12-month follow-up, the MEMO CBT intervention did not demonstrate a significant effect on reducing depressive symptoms when compared to the control, with effect sizes remaining small and statistically non-significant at both time points. |
|  |  |  |  |  |  |  | Active/ attention Control MEMO :429 |  |  | Not mentioned |  |  | Self-rated depressive symptoms (RAD-S) |  | NSS |  |
| 11 | Saulsberry et al 2013,  USA | 12 | 83 | 57 | 17.5 mean age | clinics | CATCH-IT + MI: 43 | online | Trained Physicians, Master’s level social workers/psychologists | Yes | 9 Weeks | 1.5 Months 12 Months | CESD-10 | ATQ-R PSS-fr PSS-fa SES MS AS | NSS | From post-intervention to the 12-month follow-up, the CATCH-IT MI intervention performed similarly to BA in reducing depression and suicidal tendencies among teens. Both interventions showed comparable effectiveness, with no significant differences in outcomes. The small effect sizes and confidence intervals that spanned zero suggested that neither approach was superio |
|  |  |  |  |  |  |  |  |  |  |  |  |  | Depressive disorder  (PHQ-A) |  | NSS |  |
|  |  |  |  |  |  |  | Active control CATCH-IT + BA: 40 | F2F |  | _ |  |  | PHQ-A self-harm risk |  | NSS |  |
| 12 | Stallard et al. 2013, UK | 11 | 5030 | 49 | 12 to 16 | School | Classroom-based CBT- RAP UK Programme:1753 | F2F | 39 facilitators external to the school | Yes | 11 weeks nine sessions, two boosters each approximately 50–60 minutes | 6 Months 12 Months | Depression (SMFQ27) | RCAD CATS SCS AQS IRB EQ-5D | NSS | The absence of statistically significant differences among the groups at both the 6-month and 12-month follow-ups suggests that the RAP-UK- classroom-based CBT and AC-PSHE did not outperform the usual PSHE ( Control) in reducing depressive symptoms. There is a trend suggesting that usual PSHE might be more beneficial than the intervention and active control than usual PHSE by school |
|  |  |  |  |  |  |  | Attention control Personal, Social and Health Education:1673 |  | 35 External facilitators & Staff members | No |  |  |  |  |  |  |
|  |  |  |  |  |  |  |  |  |  |  |  |  |  |  | NSS |  |
|  |  |  |  |  |  |  | Active Control: 1604 |  | 35 Staff members trained for the intervention delivery |  |  |  |  |  |  |  |
| 13 | Merry et al 2012, New Zealand | 11 | 187 | 63 | 12 -19 | School & Clinic | SPARX:94 | Computer-  based (CD–ROM) | Research assistants | Not mentioned | 4 to 7 Weeks | 2 Months 5 months | Depression  (CDRS-R) | RADS-2 MFQ PQ-LES-MFQ Kazdin HPLS SCAS | SS | The SPARX treatment can be considered no worse than the control treatment and potentially even better (though not statistically proven to be better, as the CI includes values both less than and greater than zero, and the study was designed to test non-inferiority, |
|  |  |  |  |  |  |  | Control/ TAU:93 | F2F | - | - |  |  |  |  |  |  |

**Legends:** 1. Brief Behavioral Therapy (BBT);Assisted Referral to Care (ARC);Mood and Feelings Questionnaire [MFQ]; Screen for Anxiety Related Emotional Disorders [SCARED], Cognitive Behaviour Therapy [CBT ]; Depression Self-Rating Scale for Children (DSRS-C); Pediatric Quality of Life Inventory ( PHQ-9); Strengths and Difficulties Questioner - Parents (SDQ); The Adolescent Health Promotion Short Form (AHP-SF);The Rosenberg Self-Esteem Scale (RSES);The Trait Emotional Intelligence Questionnaire–Adolescent; Short Form (TEIQue-ASF); ABFT= Attachment Based Family Therapy; Empowering a Multimodal Pathway Toward Healthy Youth (EMPATHY); CDI = Children’s Depression Inventory; CDRS-R = Children’s Depression Rating Scale-Revised; SIQ-Jr = Suicidal Ideation Questionnaire-Junior; CGAS = Children-Global Assessment Scale; BFAM; Brief Family Assessment Measure; FEICS = Family Emotional Involvement and Criticism Scale; BDI = Beck Depression Inventory; Int= Intervention; Cont= Control; SS, Statistically Significant; NSS, Not Statistically Significant; Smart, Positive, Active, Realistic, X-factor (SPARX); TAU, treatment as usual; Competent Adulthood Transition with Cognitive-behavioral, Humanistic and Interpersonal Training Internet site + Motivational interview (CATCH-IT+ (MI) Brief advice ( BA); The Resourceful Adolescent Programme (RAP) RAP a class room based CBT, cognitive–behavioral therapy; AC+PSHE, Attention Control + Personal, Social and Health Education ( AC+ PSHE); Multimedia Mobile Phone Programme (MEMO); an internet-based CBT program (Reframe-IT); TEPSI, Talleres de Education Psychological ( for parents); teen Mental Health First Aid (tMHFA); Physical First Aid (PFA); **TOOLs:** SCDRS-R=children’s depression rating scale-revised; RADS-2=Reynolds adolescent depression scale— second edition; MFQ=mood and feelings questionnaire; PQ-LES-Q= pediatric quality of life enjoyment and satisfaction questionnaire; Kazdin HPLS=Kazdin; Hopelessness Scale for Children; SCAS=Spence Children’s Anxiety Scale. Center for Epidemiologic Studies Depression (CES-D, 10 item scale) = The Patient Health Questionnaire-Adolescent (PHQ-A)= Diagnostic and Statistical Manual of Mental Disorders – Fourth Edition, Text Revision (DSM-IV-TR)= Automatic Negative Thoughts-Revised (ATQ-R)= Perceived Support from Friends (PSS-fr) Perceived Support from Family (PSS-fa) Self-efficacy Scale (SES); Motivation Scale (MS); Attitudes Scale (AS); Short Mood and Feelings Questionnaire (SMFQ – 27 item); Youth Risk Behaviour Survey (YRBS) Kiddie-Schedule for Affective Disorders and Schizophrenia (K-SADS); Suicidal Ideation Questionnaire (SIQ); Beck Hopelessness Scale (BHS); Multidimensional Anxiety Scale for Children (MASC); Negative Problem-Oriented Questionnaire (NPOQ); Cognitive-Behavioral Therapy Skills Questionnaire (CBTSQ); Children’s Depression Inventory – (CDI); Diagnostic Interview Schedule for Children –(DISC-IV); Beck Depression Inventory-R – (BDI-R); Family Emotional Involvement and Criticism Scale – (FEICS); Brief Family Assessment Measure – ( B-FAM); Families Scale (FS); Kessler Psychological Distress Scale (KPDS)
