# Supplementary material for: Effectiveness of preventive interventions on adolescents’ depression and suicidal tendency: a systematic review of randomized controlled trials
Source: Front Psychol. 2025 May 5;16:1356816. doi: 10.3389/fpsyg.2025.1356816 (PMC12087179; doi:10.3389/fpsyg.2025.1356816)
Supplement: Supplementary file 3 [file Supplementary_file_2.docx]

| Supplementary File 2: Study Inclusion and Exclusion Criteria. | | |
| --- | --- | --- |
| **Sr. no** | **Category** | **Inclusion Criterion** |
| 1 | Year of publication | January 2011- December 2023 |
| 2 | Language | English |
| 3 | Outcome Measures | Reduction in depression rates  Reduction in Suicide ideation/ thoughts |
| 4 | Age | 11-19 |
| 5 | Setting | 1. Community 2. School |
| 6 | Methodology | RCT |
| 7 | Tool | Studies with valid and reliable tools to screen depression and suicidal ideation among teens |
| **Sr. no** |  | **Inclusion Criterion** |
| 1 | Year of publication | Before January 2011 |
| 2 | Language | Other than English |
| 3 | Outcome Measures | Post COVID-19 depression, psychotic depression, |
| 4 | Setting | In patients / Hospital |
| 5 | Methodology | RCT but Pilot  RCT but Study protocol |
