# Supplementary material for: Effectiveness of preventive interventions on adolescents’ depression and suicidal tendency: a systematic review of randomized controlled trials
Source: Front Psychol. 2025 May 5;16:1356816. doi: 10.3389/fpsyg.2025.1356816 (PMC12087179; doi:10.3389/fpsyg.2025.1356816)
Supplement: Supplementary file 4 [file Supplementary_file_3.docx]

**Supplementary File 3**

**Search strategy**

- MESH term/Key words – Please refer the table below.

**Advanced search focus:**

- individual versus group based, school based versus community based

# of Total Original References 1 Total # of References: 1093 (January 2011- 15 December 2023)

PubMed = 418

CINAHL = 279

APA PsycInfo = 309

Embase = 87

# of Duplicates Removed: 254

# of References Screened: **839**

*Revised Search ( December 16 2023- February 23 2024)

# of Total New References 3: 117

PubMed = 72

CINAHL = 07

APA PsycInfo = 30

Embase = 08

# of Duplicates Removed: 25

# of New References to Screen: 92

For PRISMA flow diagram:

**Database: PubMed**

| Set # |  | Results |
| --- | --- | --- |
| 1 | ("Pediatrics"[Mesh] OR pediatric OR pediatrics OR paediatric OR paediatrics OR juvenile OR juveniles OR "Child"[Mesh] OR child OR children OR childhood OR girl OR girls OR boy OR boys OR preadolescent OR preadolescents OR prepubescent OR "Adolescent"[Mesh] OR adolescent OR adolescents OR adolescence OR youth OR youths OR “young adults” OR “young people” OR teenager OR teenagers OR teenaged OR teenage OR teen OR teens) NOT ("Adult"[Mesh] NOT ("Adolescent"[Mesh] OR "Child"[Mesh])) |  |
| 2 | "Psychotherapy"[Mesh] OR “Mental health intervention” OR “mental health interventions” OR “Psychological Therapy” OR “psychological therapies” OR non-pharmacological OR non-pharmacotherapy OR “psychological intervention” Or “Psychological interventions” OR “psychosocial Intervention” OR “psychosocial interventions” OR psychotherapy OR psychotherapies |  |
| 3 | Depression[MeSH] OR “depressive disorder”[MeSH] OR “Uni-polar affective Disorder” OR depression OR depressive OR depressed |  |
| 4 | Suicide[MeSH] OR "Self-Injurious Behavior"[Mesh] OR Suicide OR Suicides OR suicidal OR suicidality OR self-harm OR self-injury OR self-injurious OR “self harm” OR “self injury” OR “self injurious” OR “self-destructive behavior” OR “self-destructive behaviors” |  |
| 5 | #1 AND #2 AND #3 AND #4 |  |
| 6 | (("randomized controlled trial"[Publication Type] OR "controlled clinical trial"[Publication Type] OR "randomized"[Title/Abstract] OR "placebo"[Title/Abstract] OR "drug therapy"[MeSH Subheading] OR "randomly"[Title/Abstract] OR "trial"[Title/Abstract] OR "groups"[Title/Abstract]) NOT ("animals"[MeSH Terms] NOT "humans"[MeSH Terms])) |  |
| 7 | #5 AND #6 |  |
| 8 | #7 AND English[lang] |  |
| 9 | #8 AND (("2011"[Date - Publication] : "3000"[Date - Publication])) | 418/*72 |

**CINAHL**

| Set # |  | Results |
| --- | --- | --- |
| 1 | MH "Pediatrics+" OR MH "Child+" OR MH "Adolescence+" OR pediatric OR pediatrics OR paediatric OR paediatrics OR juvenile OR juveniles OR child OR children OR childhood OR girl OR girls OR boy OR boys OR preadolescent OR preadolescents OR prepubescent OR adolescent OR adolescents OR adolescence OR youth OR youths OR “young adults” OR “young people” OR teenager OR teenagers OR teenaged OR teenage OR teen OR teens |  |
| 2 | MH "Psychotherapy+" OR “Mental health intervention” OR “mental health interventions” OR “Psychological Therapy” OR “psychological therapies” OR non-pharmacological OR non-pharmacotherapy OR “psychological intervention” Or “Psychological interventions” OR “psychosocial Intervention” OR “psychosocial interventions” OR psychotherapy OR psychotherapies |  |
| 3 | MH "Depression+" OR “Uni-polar affective Disorder” OR depression OR depressive OR depressed |  |
| 4 | MH "Suicide+" OR MH "Self-Injurious Behavior" OR Suicide OR Suicides OR suicidal OR suicidality OR self-harm OR self-injury OR self-injurious OR “self harm” OR “self injury” OR “self injurious” OR “self-destructive behavior” OR “self-destructive behaviors” |  |
| 5 | #1 AND #2 AND #3 AND #4 |  |
| 6 | MH "Randomized Controlled Trials" OR "controlled clinical trial" OR "randomized" OR "randomised" OR "randomization" OR "randomisation" OR "placebo" OR "randomly" OR "trial" OR groups |  |
| 7 | #5 AND #6 |  |
| 8 | #7 AND Narrow by Language: - english |  |
| 9 | #8 AND Limiters - Publication Date: 20110101-20231231 | 279/07 |

**APA PsycInfo**

| Set # |  | Results |
| --- | --- | --- |
| 1 | pediatric OR pediatrics OR paediatric OR paediatrics OR juvenile OR juveniles OR child OR children OR childhood OR girl OR girls OR boy OR boys OR preadolescent OR preadolescents OR prepubescent OR adolescent OR adolescents OR adolescence OR youth OR youths OR “young adults” OR “young people” OR teenager OR teenagers OR teenaged OR teenage OR teen OR teens |  |
| 2 | DE "Psychotherapy" OR DE "Adlerian Psychotherapy" OR DE "Adolescent Psychotherapy" OR DE "Affirmative Therapy" OR DE "Analytical Psychotherapy" OR DE "Autogenic Training" OR DE "Brief Psychotherapy" OR DE "Brief Relational Therapy" OR DE "Child Psychotherapy" OR DE "Client Centered Therapy" OR DE "Conversion Therapy" OR DE "Couples Therapy" OR DE "Eclectic Psychotherapy" OR DE "Emotion Focused Therapy" OR DE "Existential Therapy" OR DE "Experiential Psychotherapy" OR DE "Expressive Psychotherapy" OR DE "Eye Movement Desensitization Therapy" OR DE "Feminist Therapy" OR DE "Geriatric Psychotherapy" OR DE "Gestalt Therapy" OR DE "Group Psychotherapy" OR DE "Guided Imagery" OR DE "Humanistic Psychotherapy" OR DE "Hypnotherapy" OR DE "Individual Psychotherapy" OR DE "Insight Therapy" OR DE "Integrative Psychotherapy" OR DE "Interpersonal Psychotherapy" OR DE "Logotherapy" OR DE "Narrative Therapy" OR DE "Network Therapy" OR DE "Persuasion Therapy" OR DE "Primal Therapy" OR DE "Psychoanalysis" OR DE "Psychodrama" OR DE "Psychodynamic Psychotherapy" OR DE "Psychotherapeutic Counseling" OR DE "Psychotherapeutic Techniques" OR DE "Rational Emotive Behavior Therapy" OR DE "Reality Therapy" OR DE "Relationship Therapy" OR DE "Solution Focused Therapy" OR DE "Strategic Therapy" OR DE "Supportive Psychotherapy" OR DE "Transactional Analysis" OR DE "Mental Health Programs" OR DE "Crisis Intervention Services" OR DE "Deinstitutionalization" OR DE "Home Visiting Programs" OR DE "Hot Line Services" OR DE "Suicide Prevention Centers" OR DE "Psychoeducation" OR DE "Self-Help Techniques" OR DE "Self-Management" OR “Mental health intervention” OR “mental health interventions” OR “Psychological Therapy” OR “psychological therapies” OR non-pharmacological OR non-pharmacotherapy OR “psychological intervention” Or “Psychological interventions” OR “psychosocial Intervention” OR “psychosocial interventions” OR psychotherapy OR psychotherapies |  |
| 3 | DE "Major Depression" OR DE "Anaclitic Depression" OR DE "Dysthymic Disorder" OR DE "Endogenous Depression" OR DE "Late Life Depression" OR DE "Postpartum Depression" OR DE "Reactive Depression" OR DE "Recurrent Depression" OR DE "Treatment Resistant Depression" OR “Uni-polar affective Disorder” OR depression OR depressive OR depressed |  |
| 4 | DE "Suicide" OR DE "Military Suicide" OR DE "Youth Suicide" OR DE "Suicide Prevention" OR DE "Self-Injurious Behavior" OR DE "Head Banging" OR DE "Self-Inflicted Wounds" OR DE "Self-Mutilation" OR DE "Self-Poisoning" OR Suicide OR Suicides OR suicidal OR suicidality OR self-harm OR self-injury OR self-injurious OR “self harm” OR “self injury” OR “self injurious” OR “self-destructive behavior” OR “self-destructive behaviors” |  |
| 5 | #1 AND #2 AND #3 AND #4 |  |
| 6 | ZC "treatment outcome/clinical trial" OR DE "Clinical Trials OR "controlled clinical trial" OR "randomized" OR "randomised" OR "randomization" OR "randomisation" OR "placebo" OR "randomly" OR "trial" OR groups |  |
| 7 | #5 AND #6 |  |
| 8 | #7 AND Narrow by Language: - english |  |
| 9 | #8 AND Limiters - Publication Year: 2011- |  |
| 10 | #9 AND Source Types - Academic Journals | 309/30 |

**Embase**

| Set # |  | Results |
| --- | --- | --- |
| 1 | 'pediatrics'/exp OR 'child'/exp OR 'adolescent'/exp OR pediatric OR pediatrics OR paediatric OR paediatrics OR juvenile OR juveniles OR child OR children OR childhood OR girl OR girls OR boy OR boys OR preadolescent OR preadolescents OR prepubescent OR adolescent OR adolescents OR adolescence OR youth OR youths OR “young adults” OR “young people” OR teenager OR teenagers OR teenaged OR teenage OR teen OR teens |  |
| 2 | 'psychotherapy'/exp OR ‘Mental health intervention’ OR ‘mental health interventions’ OR ‘Psychological Therapy’ OR ‘psychological therapies’ OR non-pharmacological OR non-pharmacotherapy OR ‘psychological intervention’ Or ‘Psychological interventions’ OR ‘psychosocial Intervention’ OR ‘psychosocial interventions’ OR psychotherapy OR psychotherapies |  |
| 3 | 'depression'/exp OR ‘Uni-polar affective Disorder’ OR depression OR depressive OR depressed |  |
| 4 | 'suicide'/exp OR 'automutilation'/exp OR Suicide OR Suicides OR suicidal OR suicidality OR self-harm OR self-injury OR self-injurious OR ‘self harm’ OR ‘self injury’ OR ‘self injurious’ OR ‘self-destructive behavior’ OR ‘self-destructive behaviors’ |  |
| 5 | #1 AND #2 AND #3 AND #4 |  |
| 6 | 'randomized controlled trial'/exp OR 'crossover procedure'/exp OR 'double blind procedure'/exp OR 'single blind procedure'/exp OR random* OR factorial* OR crossover* OR cross NEAR/1 over* OR placebo* OR doubl* NEAR/1 blind* OR singl* NEAR/1 blind* OR assign* OR allocat* OR volunteer* |  |
| 7 | #5 AND #6 |  |
| 8 | #7 AND [english]/lim |  |
| 9 | #8 AND [2011-2023]/py |  |
| 10 | #9 AND [embase]/lim NOT ([embase]/lim AND [medline]/lim) |  |
| 11 | #10 AND ('article'/it OR 'article in press'/it OR 'preprint'/it) | 87/08 |

**PakMediNet: cannot get this one to work with the Advanced Search**

**I also could not get this one to work, and I tried simplifying the search a lot.**

| Set # |  | Results |
| --- | --- | --- |
| 1 | pediatric OR pediatrics OR paediatric OR paediatrics OR juvenile OR juveniles OR child OR children OR childhood OR girl OR girls OR boy OR boys OR preadolescent OR preadolescents OR prepubescent OR adolescent OR adolescents OR adolescence OR youth OR youths OR “young adults” OR “young people” OR teenager OR teenagers OR teenaged OR teenage OR teen OR teens |  |
| 2 | “Mental health intervention” OR “mental health interventions” OR “Psychological Therapy” OR “psychological therapies” OR non-pharmacological OR non-pharmacotherapy OR “psychological intervention” Or “Psychological interventions” OR “psychosocial Intervention” OR “psychosocial interventions” OR psychotherapy OR psychotherapies |  |
| 3 | “Uni-polar affective Disorder” OR depression OR depressive OR depressed |  |
| 4 | Suicide OR Suicides OR suicidal OR suicidality OR self-harm OR self-injury OR self-injurious OR “self harm” OR “self injury” OR “self injurious” OR “self-destructive behavior” OR “self-destructive behaviors” |  |
| 5 | randomized OR randomised OR randomization OR randomisation OR randomly OR trial OR trials |  |
| 6 | #1 AND #2 AND #3 AND #4 |  |
| 7 | #5 AND Narrow by Language: - English |  |
| 8 | #6 AND Limiters - Publication Year: 2011-2024 | 00/00 |
