# Supplementary material for: Effectiveness of preventive interventions on adolescents’ depression and suicidal tendency: a systematic review of randomized controlled trials
Source: Front Psychol. 2025 May 5;16:1356816. doi: 10.3389/fpsyg.2025.1356816 (PMC12087179; doi:10.3389/fpsyg.2025.1356816)
Supplement: Supplementary file 5 [file Supplementary_file_4.docx]

Supplementary file 4:JBI appraisal checklist

Table 2. Summary of RCTs study appraisal based on JBI appraisal checklist

| Sr # | Author & year | JBI critical appraisal checklist questions & answers | | | | | | | | | | | | | Over all Appraisal of 13 |
| --- | --- | --- | --- | --- | --- | --- | --- | --- | --- | --- | --- | --- | --- | --- | --- |
|  |  | 1 | 2 | 3 | 4 | 5 | 6 | 7 | 8 | 9 | 10 | 11 | 12 | 13 |  |
| 1. 1 | Schwartz et al., 2023 | Y | Y | Y | Y | N | Y | N | Y | Y | Y | Y | Y | Y | 11 |
|  | Nagamitsu et al., 2022 | Y | Y | Y | U | U | Y | Y | Y | Y | Y | Y | Y | Y | 11 |
|  | Waraan et al., 2021^a^ | Y | Y | Y | Y | U | U | U | Y | Y | Y | Y | Y | Y | 10 |
|  | Waraan et al., 2021b | Y | Y | Y | Y | U | U | U | Y | Y | Y | Y | Y | Y | 10 |
|  | Jonge-Heesen et al., 2020  Netherlands | Y | Y | Y | N | U | N | Y | Y | Y | Y | Y | Y | Y | 10 |
|  | Diamond et al, 2019 | Y | U | Y | Y | N | Y | Y | Y | Y | Y | Y | Y | Y | 12 |
|  | Bernal, 2019 | Y | Y | Y | Y | U | U | Y | Y | Y | Y | Y | Y | Y | 11 |
|  | Silverstone et al 2017, | N | N | Y | N | N | Y | Y | Y | Y | Y | Y | Y | Y | 9 |
|  | Hetrick, 2017 | Y | Y | Y | Y | N | Y | Y | N | Y | Y | Y | Y | Y | 11 |
|  | Whittaker et al. 2017 | Y | Y | Y | Y | Y | Y | Y | Y | Y | Y | Y | Y | Y | 13 |
|  | Saulsberry et al, 2013 | Y | Y | Y | Y | N | Y | Y | Y | Y | Y | Y | Y | Y | 12 |
|  | Stallard et al, 2013 | Y | Y | Y | N | N | Y | Y | Y | Y | Y | Y | Y | Y | 11 |
|  | Merry, 2012 | Y | Y | Y | N | N | Y | Y | Y | Y | Y | Y | Y | Y | 11 |

Y=yes; N=no; U=unclear; NA=not applicable.

**JBI-RCT checklist Domains**

1. True randomization used
2. Allocation to groups concealed
3. Groups similar at baseline
4. Participants blind to treatment
5. Those delivering treatment blind
6. Outcomes assessors blind
7. Groups treated identically
8. Follow up complete
9. Participants analyzed as they were randomized
10. Outcomes measured equally for groups
11. Outcomes measured in a reliable way
12. Appropriate statistical analysis used
13. Not Any deviations found from the standard RCT
